# Brodifacoum Levels and Biomarkers in Coastal Fish Species following a Rodent Eradication in an Italian Marine Protected Area: Preliminary Results

**DOI:** 10.3390/life13020415

**Published:** 2023-02-02

**Authors:** Ilaria Caliani, Agata Di Noi, Carlo Amico, Roberto Berni, Marco Romi, Giampiero Cai, Massimo Guarnieri, Augusto Navone, Giovanna Spano, Gregg R. Howald, Paolo Sposimo, Letizia Marsili

**Affiliations:** 1 Department of Physical, Earth and Environmental Sciences, University of Siena, via Mattioli, 4, 53100 Siena, Italy; 2 Department of Life Sciences, University of Siena, via Mattioli, 4, 53100 Siena, Italy; 3 Luxembourg Institute of Science and Technology (LIST), Environmental Research and Innovation (ERIN) Department, L-4940 Hautcharage, Luxembourg; 4 Tavolara A.M.P., Punta Coda Cavallo, via Dante, 1, 07026 Olbia, Italy; 5 Advanced Conservation Strategies, 2261 Success Trail, Midway, UT 84049, USA; 6 Coastal Conservation, 775 Abbington Lane, Tappen, BC V0E 2X3, Canada; 7 NEMO Ltd., Viale G. Mazzini 26, 50132 Firenze, Italy

**Keywords:** brodifacoum rodenticides, aerial broadcast, vitamin K, non-target marine species, secondary exposure, DNA damage

## Abstract

Brodifacoum is the most common rodenticide used for the eradication of invasive rodents from islands. It blocks the vitamin K cycle, resulting in hemorrhages in target mammals. Non-target species may be incidentally exposed to brodifacoum, including marine species. A case study conducted on the Italian Marine Protected Area of Tavolara Island was reported after a rodent eradication using the aerial broadcast of a brodifacoum pellet. Brodifacoum presence and effects on non-target marine organisms were investigated. Different fish species were sampled, and a set of analyses was conducted to determine vitamin K and vitamin K epoxide reductase concentrations, prothrombin time, and erythrocytic nuclear abnormalities (ENA) assay. In all the examined organisms, brodifacoum was not detected. The results obtained showed differences in vitamin K and vitamin K epoxide concentrations among the samples studied, with a positive correlation for three species between vitamin K, vitamin K epoxide, and fish weight. The prothrombin time assay showed a good blood clotting capacity in the fish. Higher abnormality values were recorded for four species. The results of this study suggest that it is possible to hypothesize that the sampled fish were not likely to have been exposed to brodifacoum and that consequently there are no negative issues concerning human consumption.

## 1. Introduction

The anticoagulants are powerful rodenticides that block the vitamin K cycle and prevent the synthesis of active forms of different blood-clotting factors (II, VII, IX, and X) in the liver necessary for hemostasis. Mammalian and avian’ exposure to these substances can lead to death due to internal bleeding [1,2]. First-generation anticoagulant rodenticides (FGARs) such as warfarin, chlorophacinone, and diphacinone, were first introduced in the late 1940s. They require multiple exposure event over timeto cause rodent death. Their widespread and long term use has caused genetic resistance in rats and mice [1,3]. Second-generation anticoagulant rodenticides (SGARs) such as brodifacoum, difenacoum, difethialone, and flocoumafen were developed to overcome resistance in rats and mice and can be toxic after a single feed on some bait formulations. The SGARs are much more effective, but persistent and can be bioaccumulative compared to FGARs [3,4,5].

The vitamin K epoxide reductase (VKOR) enzyme is involved in the oxidation/reduction cycle involving vitamin K in the liver. VKOR recycles vitamin K epoxide in vitamin K that is reduced in vitamin K hydroquinone. It is a cofactor in carboxylation, which produces γ-carboxyglutamic acid (Gla) and activates the blood coagulation factors prothrombin (factor II) VII, IX, and X, plasma proteins C, M, S, Z, and protein osteocalcin, and the Gla protein of the matrix at the bone level. Simultaneously, vitamin K hydroquinone is oxidized to vitamin K epoxide [6,7]. The SGARs inactivate VKOR by binding it, preventing the oxidation/reduction cycle and inactive clotting factors, resulting in hemorrhages in susceptible birds and mammals [2,7].

Brodifacoum is the most common rodenticide used in rodent eradication. It is persistent in soil with a half-life time (t½) of 157 days and is a compound relatively insoluble in water [8]. The latent period in rodents is typically 3–7 days after exposure to a lethal dose [9]. Brodifacoum is relatively persistent in mammals (150–200 days); particularly in the liver and to a lesser extent in muscles [10].

Although the use of anticoagulant rodenticides is an effective means for achieving the eradication of invasive rodents, these compounds are responsible for many unintentional poisoning of non-target organisms, particularly birds and mammals. Exposure can occur through the ingestion of baits (primary exposure) or ingestion of other animals containing residual rodenticide concentrations (secondary exposure) [11]. In particular, secondary poisoning is most commonly associated with SGARs [12,13,14,15], likely reflective of their persistence and widespread use for rodent control. The SGARs have been detected in invertebrates [16] and in a diverse number of species of carnivorous mammals, granivorous birds, and diurnal and nocturnal birds of prey [17]. Moreover, Wiens et al. (2019) [18] observed the presence of anticoagulant rodenticides in 48% of the barred owls (*Strix varia*) sampled in two different American states and, among the different rodenticides, brodifacoum was the most likely to be detected, reflecting its widespread use internationally.

It is believed that marine species are at low risk of exposure to brodifacoum as bait application into the marine environment is incidental, and bait pellets are rapidly decomposed in water with low quantities of brodifacoum typically reaching the marine environment [19,20]. However, where residues enter the marine environment, it is possible that residues can transfer and accumulate in the tissues of marine species and potentially be consumed by people [21,22]. Relatively few studies investigated the rodenticides’ presence in non-target marine vertebrates [20,23]. Brodifacoum residues have been confirmed to enter the marine environments after bait application targeting rodents on islands, including blue cod (*Parapercis colias*), mussel (*Mytilus edulis*), and limpet (*Cellana ornata*) specimens from Ulva Island (New Zealand) [22] and in square-tailed mullet (*Liza vaigiensis*) from Palmyra Atoll (tropical Pacific) [24]. Siers et al. (2020) detected low levels of brodifacoum in two individuals of blacktail snapper (*Lutjanus fulvus*) three years after the application of the rodenticide on Wake Island (Pacific Ocean) [25].

A thorough analysis of the acquired relevant literature and data yielded that contamination caused by treatment with rodenticides, specifically brodifacoum, has not been previously investigated in terms of the biological effects on vitamin K and vitamin K epoxide, and/or DNA damage.

The aim of this study was to investigate brodifacoum presence and its effects on different non-target marine species. To this purpose, different fish species were sampled in Tavolara Island (Sardinia), an Italian Marine Protected Area (MPA), after a rat eradication with aerial broadcasting of pellets containing 50 ppm brodifacoum. The risk of secondary poisoning to humans who may consume these marine species was also evaluated.

## 2. Materials and Methods

### 2.1. Chemicals and Reagents

Dichloromethane, acetone, methanol, acetonitrile, sucrose buffer, KCl buffer, isopropanol, hexane, and Giemsa stain were purchased from Sigma-Aldrich, St. Louis, MO, USA. Standard solutions of brodifacoum, vitamin K, and vitamin K 2,3-epoxide were also purchased from Sigma-Aldrich, St. Louis, MO, USA. The ReadiPlasTin kit (HemosiIL) was purchased from Werfen, Milan, Italy.

### 2.2. Study Site: Tavolara Island (Sardinia, Italy)

**Figure 1:**
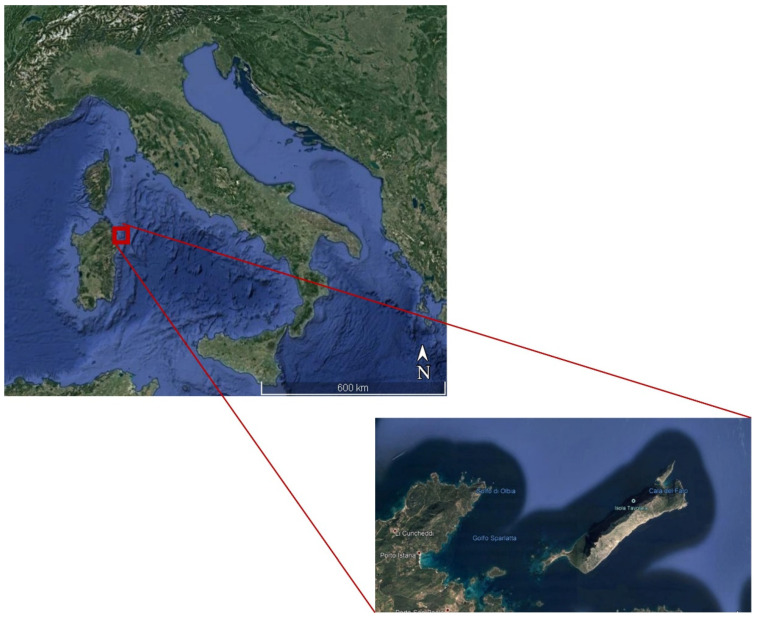
Map of Tavolara Island showing the sampling area.

The Tavolara Punta Coda Cavallo MPA, established in 1997 by decree of the Italian Ministry of Environment, includes approximately 15,000 hectares of sea and the coastal territories of Olbia, Loiri Porto San Paolo, and San Teodoro municipalities. In 2007, the MPA obtained the recognition of SPAMI (Specially Protected Area of Mediterranean Importance), thanks to the naturalistic values it contains, and includes, in its territory, Tavolara Island (Figure 1). This island is 6 km long, with a maximum width of 1.5 km and a flat surface area of about 600 hectares. It comprises a main body, with a single ridgeline all above 500 m and a maximum elevation of 565 m, and two promontories where there are small human settlements.

Tavolara hosts many species protected by regional regulations and international conventions, such as the Mediterranean shag (*Phalacrocorax aristotelis desmarestii*), Cory’s shearwater (*Calonectris diomedea*), Audoin’s gull (*Larus audouinii*), and yelkouan shearwater (*Puffinus yelkouan*). The MPA is inhabited by 30–50% of the world’s population of yelkouan shearwater, classified as vulnerable at global level. One of the most serious threats to the yelkouan shearwater is the predation of eggs and chicks by rats. In the period between 2006 and 2011, almost all the monitored nests on Tavolara Island were lost to rat predation, except for the very few located in inaccessible caves and those present in areas subject to local rat suppression prior to the eradication. Although there is a paucity of data specific to Tavolara Island, rats may also predate other species, such as lizards and geckos, bats, and shrews, altering the entire ecosystem.

### 2.3. Bait Application Method

Due to the island’s topography, the eradication of rodents from Tavolara was achieved through the aerial broadcast of a 50 ppm brodifacoum pelleted bait. According to the current best practices on rodents eradication using aerial baiting [26], two separate bait applications, about 20 days apart, had been carried out from October to November 2017. The application of bait was completed by a helicopter with a sowing bucket, along parallel flight lines guided by GPS. The sowing rate was 12.5 kg/ha, calculated on the ground for the first baiting and 6.5 kg/ha for the second one. The bait used was Brocum™, a cereal-based pellet containing 50 ppm brodifacoum. The inhabited areas had been excluded from aerial baiting and treated on the ground, with rodenticide bait contained in bait stations.

Tavolara’s topography, with its steep rocky coastline, makes it almost impossible to prevent the pellets from rolling into the sea. For this reason, preliminary tests were conducted in the coastal waters of the island, to identify the fish species most at risk of ingesting the pellets, and tests were carried out during aerial distribution to estimate the number of pellets that rolled into the sea. Based on this latest survey, the average quantity of bait dropped into the sea, within 5 m of the coast in the only sections with cliffs, was estimated to be equal to 1.6 g/m^2^ (Guidetti and Bussotti, unpublished data).

### 2.4. Fish Sampling

In November 2017, ten days post-baiting for the second application, eight different fish species, in the non-reproductive period, were sampled near the coastline using manual fishing with baited hooks rods and gill nets. Several morphological parameters (length and weight) of the animals were noted down. Blood samples were collected from the caudal vein, using a disposable heparinized syringe, and transferred into sterile tubes. For each animal, a drop of blood was used to prepare the blood smears and the rest of the blood was centrifuged at 5000× *g* for 5 min. The obtained plasma was transferred into labelled tubes containing approximately 5 µL of antiproteases and then immediately stored at − 80 °C. After the sacrifice of the animals, the liver was collected and stored at −80 °C.

### 2.5. Brodifacoum Analysis

Analysis of brodifacoum residues was performed by an HPLC/fluorescence system in liver tissue, following Christensen et al. (2012) [27] with modifications. Samples were lyophilized in an Edwards freeze drier for 2 days, homogenized with potter, and then 0.4 g was extracted with 10 mL of dichloromethane/acetone mixture (70/30 *v*/*v*) and 3 mL of methanol. Extraction solvent, 13 mL, was mixed thoroughly with the homogenized tissue and shaken for 5 min, then centrifuged for 15 min at 20 °C at 800× *g*. The supernatant was collected and transferred. The residue was resuspended with 2 mL of methanol, shaken for 5 min, and centrifuged for 15 min at 20 °C at 800× *g*. The new supernatant obtained was collected and combined with the previous one. A sample purification was performed twice with liquid chromatography using a dichloromethane/acetone mixture (70/30 *v/v*) on a column containing sodio-phosphate (5 g). The eluate was evaporated to dryness and subsequently redissolved in 1 mL methanol and analyzed by HPLC analysis (Waters™ HPLC system: 600 pump) using a reversed-phase column (54851 SUPELCO SUPELCOSIL™ LC-18-DB (25 cm × 21.2 mm)) and an acetonitrile/water as the gradient. The initial gradient was 100% acetonitrile, reaching 0% acetonitrile and 100% water after 15 min, then remaining stable for 5 min (run time: 20 min). The volume injected was 20 μL and the flow rate was 0.50 mL/min. The external standard consisted of PESTANAL^®^ analytical standards. Assay reproducibility was determined by five repeated analyses (variation coefficient was in the range of 1–3%), recoveries of standard ranged 80–98%, and the absence of brodifacoum in the blanks.

### 2.6. Vitamin K and Vitamin K Epoxide Reductase Analyses

The levels of vitamin K in the liver were measured in the microsomal fraction using the Sherman and Sander method (1981) [28]. To obtain the microsomal fraction, the liver was homogenized in a sucrose buffer 0.25 M (pH 7.5) (ratio 1:4 g/mL) using a Tissue grinder (Ultra-Turrax^®^ T25 basic IKA, Saint Louis, MS, USA) at 4 °C. The homogenate was centrifuged at 9000× *g* for 20 min and the supernatant was discarded. The pellet was centrifuged again at 100,000× *g* for 60 min. The supernatant was discarded again, and the resulting pellet was resuspended with KCl buffer 1.15% at pH 7.5 (ratio 1:2.6 g/mL) and then re-homogenized. A liquid solution of vitamin K epoxide (10 µL of vitamin K epoxide in methanol 95%) was added to 500 µL of microsomes, giving a final concentration of 10 µM. Then 1 mL of isopropanol/hexane (3:2) was added to the mixture and centrifuged at 9000× *g* for 5 min. An aliquot of 300 µL of the upper hexane phase was removed from the mixture and the remainder was left to evaporate at room temperature. The sample was diluted in 100 µL of isopropanol and stirred for 20 min. Finally, 20 µL were taken and then analyzed with HPLC. Three replicates were performed for each sample. The HPLC method was optimized following the Jakob and Elmadfa (1996) [29] method. The analysis was carried out with an RP-C18 column (SUPELCO Kromasil 100A-5u-C18 4.6 mm × 250 mm), at a flow rate of 1 mL/min and the absorbance set at 254 nm. The total run time was 15 min. The mobile phase consisted of methanol/dichloromethane 75/25% (*v*/*v*) maintained in isocratic conditions. An external standard calibration curve consisting of six points at the increasing concentrations of 1, 5, 10, 50, 150, and 300 μg per mL was prepared using the standard solution of vitamin K and vitamin K 2, 3-epoxide (vitamin K and vitamin K 2,3-epoxide LOD = 0.01 µg/mL; LOQ = 0.01 µg/mL) (Figure S1).

### 2.7. Prothrombin Time

The prothrombin time was investigated using the commercial ReadiPlasTin kit, according to the manufacturer’s instructions. Results were expressed as activity % compared to the instrument blank.

### 2.8. ENA Assay

The erythrocytic nuclear abnormalities (ENA) were investigated using the Pacheco and Santos (1997) [30] method. The blood smears were fixed with methanol for 10 min and stained with Giemsa (5%) for 30 min. For each sample, only mature erythrocytes were considered, i.e., those with non-granular cytoplasm and well-defined contours. There were 1000 erythrocytes that were counted under 1000× magnification (Olympus BX41), to evaluate the relative frequency of different nuclear lesions: kidney-shaped nuclei, lobed nuclei, segmented nuclei, and micronuclei. The results were expressed as the sum of frequencies ‰ for all the nuclear lesions observed.

### 2.9. Statistical Analysis

The statistical analysis was performed using IBM SPSS Statistics v19 (IBM SPSS, Chicago, IL, USA) and the normal distribution of the data was verified with a Shapiro–Wilk test and a Q–Q plot. Then a one-way ANOVA with Tukey’s post-hoc test was applied in order to identify the statistically significant differences among species (*p* < 0.05).

## 3. Results

The purpose of the study was not only the measurement of brodifacoum concentrations but also the evaluation of its effect on non-target species, an approach that has never been used before on fish.

The morphological parameters of the fish sampled at Tavolara Island are summarized in Table 1.

### 3.1. Brodifacoum Concentration

The analysis of 15 liver samples from different species (*Sciaena umbra* (*n* = 2), *Serranus scriba* (4), *Diplodus vulgaris* (4), *Serranus cabrilla* (1), *Sarpa salpa* (2), *Spondyliosoma cantharus* (1), and *Labrus merula* (1)) showed that brodifacoum was not detected (<MLOD) to a maximum area of 4.5 m Absorbance Units (mAU)*sec, while in the standard the total area of the 2 peaks was 1817.33 mAU*sec (Figure S2).

### 3.2. Vitamin K and Vitamin K Epoxide Reductase Concentrations

**Figure 2:**
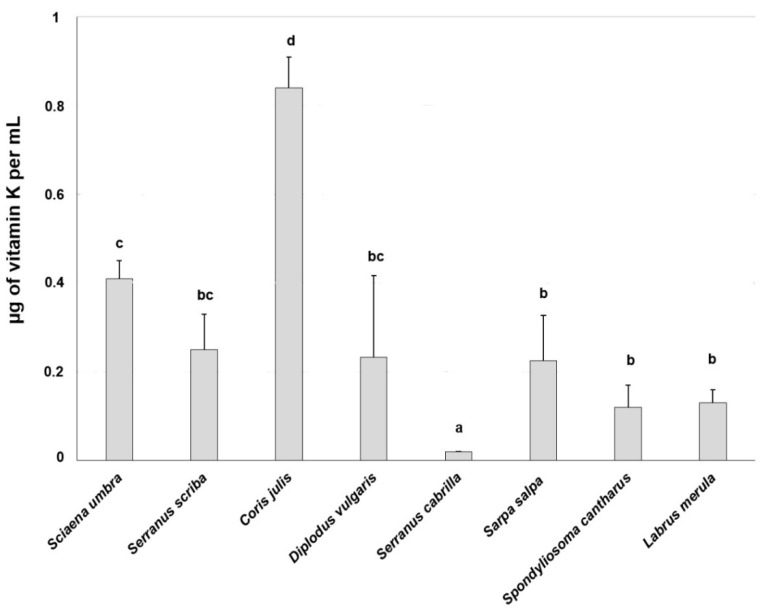
Mean ± S.D. of vitamin K total content (µg of vitamin K/mL) of microsomal hepatic fraction from different fish species sampled at Tavolara Island. Different letters indicate statistically significant differences (*p* < 0.05).

**Figure 3:**
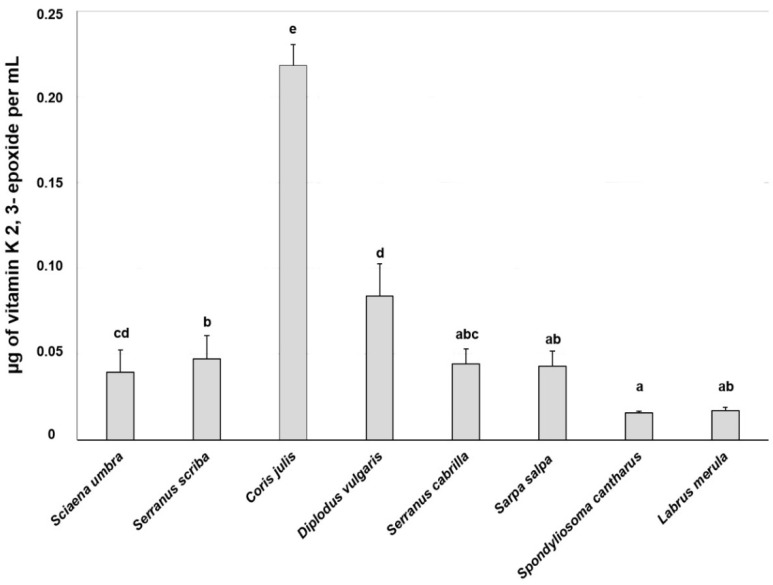
Mean ± S.D. of vitamin K 2,3-epoxide total content (µg of vitamin K epoxide/mL) of microsomal fractions from different fish species sampled at Tavolara Island. Different letters indicate statistically significant differences (*p* < 0.05).

Vitamin K and vitamin K 2,3-epoxide were evaluated in 14 samples from eight different fish species captured in waters surrounding Tavolara Island: *Sciaena umbra* (*n* = 2), *Serranus scriba* (3), *Coris julis* (1), *Diplodus vulgaris* (3), *Serranus cabrilla* (1), *Sarpa salpa* (2), *Spondyliosoma cantharus* (1), and *Labrus merula* (1). The HPLC analyses showed variations in vitamin K content, probably related to the fish species. Generally, *C. julis* showed the highest content of vitamin K, while *S. cabrilla* showed the lowest (Figure 2), with statistically significant differences compared to all the other species (*p* < 0.05). The values of vitamin K epoxide partially reflected those obtained for vitamin K. Indeed, as reported in Figure 3, *C. julis* showed the highest contents with a statistically significant difference from all the other species (*p* < 0.05), while *S. cantharus* and *L. merula*, had the lowest contents.

### 3.3. Prothrombin Time Assay

The prothrombin time assay, carried out on the plasma of the eight fish species sampled at Tavolara Island, showed results below the detection limit.

### 3.4. ENA Assay

**Figure 4:**
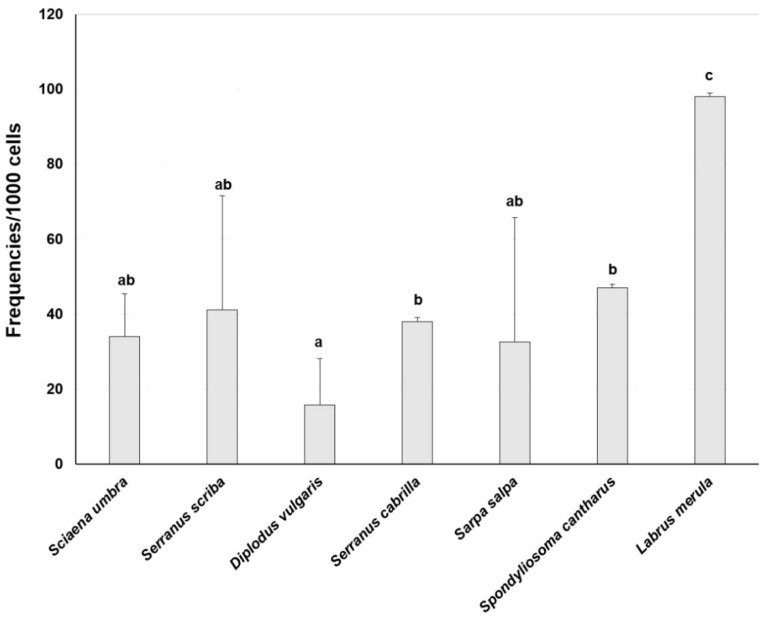
The total frequency of ENA (Mean ± S.D.) in erythrocytes of the species sampled at Tavolara Island. Different letters indicate statistically significant differences (*p* < 0.05).

Erythrocytic nuclear abnormality frequencies were evaluated in blood cells of seven fish species sampled at Tavolara Island after the rodent eradication with brodifacoum. *C. julis* was not included because of the technical difficulties in blood collection. The total frequency (‰) of abnormalities (Figure 4) in the specimens collected showed the highest values for *L. merula* (91‰) that were statistically different with respect to all the other species (*p* < 0.05). Statistical differences were observed between *D. vulgaris*, which showed the lowest values, and *S. cantharus* and *S. cabrilla* (*p* < 0.05).

Among the four different types of abnormalities, the lobed-shaped cells were the most frequent for all the investigated specimens (Table 2). Micronuclei were more abundant in *S. umbra* (1.5‰).

## 4. Discussion

The presence of brodifacoum residues and vitamin K and vitamin K 2,3-epoxide concentrations were investigated in eight different marine species from Tavolara Island. Prothrombin time and ENA assays were also evaluated. To our knowledge, this is the first study, although preliminary, that integrated chemical analysis and the evaluation of the sublethal effects for the investigation of the aerial broadcast of brodifacoum pelleted baits.

The chromatographic method allowed the evaluation of the contents of vitamin K and vitamin K epoxide from the microsomal fraction of different fish species. It is well known that after brodifacoum exposure in rats, there is a noteworthy vitamin k deficiency, due to the inhibition of vitamin K cycle, which is a direct consequence [31]. The absence of the rodenticide and the different contents of vitamin K and vitamin K epoxide found in different fish specimens suggest the hypothesis that other factors, such as the kind of species and their weight are responsible for the measured values. The obtained data in this study showed a positive correlation between three species *S. umbra*, *S. scriba*, and *S. cabrilla* and the weight (Pearson correlation values R = 0.904 for vitamin K and R = 0.753 for vitamin K epoxide). Ostermeyer and Schimdt (2001) [32] showed different levels of vitamin K in fish depending on their diet and fat mass, also reporting a relation between the fish weight and the amount of vitamin K.

Blood coagulation is the most important component of haemostasis. Beside the fact that prolonged clotting time causes mortality due to haemorrhaging, it can also constitute, in less serious situations, a change in fitness that could affect tolerance to normal physiological events and environmental stressors, such as exposure to contaminants. The measurement of plasma clotting time, such as prothrombin time, is often used as a diagnostic tool for AR intoxication in humans and house pets, while its application in captive and free-ranging animals is rare [1,33]. In general, a prothrombin time longer than 25% indicates exposure to anticoagulants [34], but it should be confirmed by the determination of AR residues in blood or in other target tissues, such as the liver and muscles. The results obtained showed a normal blood clotting capacity of sampled fish. The values obtained on common carp, *Cyprinus carpio* [35], striped sea bream (*Lithognathus mormyrus*), common dentex (*Dentex dentex*), and gilthead sea bream (*Sparus aurata*) were higher than those measured in this paper [36]. This suggests that when it comes to clotting time, assays are a sensitive, accurate, and cheap biomarker of AR exposure in field studies.

The ENA assay is a cytogenic technique that highlights alterations in erythrocyte nuclei that may lead to their fragmentation and/or micronuclei formation [37]. This test has already been used in several fish species [30,38,39,40,41,42,43,44,45] because of its simplicity, rapidity, sensitivity, and low cost. The genotoxic effect of anticoagulant rodenticides was never investigated, although there is evidence that brodifacoum can cause oxidative stress [46], which may lead to genotoxic damage. Moreover, the results obtained cannot be compared with other papers due to the lack of data concerning the frequency of abnormalities in the species used for this study. In different marine species from areas considered controls, such as *Liza aurata* [47], *Anguilla anguilla* [42,43,48], and *Sparus aurata* [49], the frequency of basal abnormalities is in the range of 3–20‰. These values are lower compared to the ENA frequencies of most of the species evaluated in this study. These modifications in the percentage of abnormalities could be due to genotoxic environmental contaminants that could be present in the studied area. On the other hand, these results could be due to other causes; in fact, some chromosome responses may also be linked to seasonal differences, such as water temperature as Gusso-Choueri and fellow researchers (2016) [50] reported. Future studies are needed to investigate the baseline values of these species’ nuclear abnormalities in greater depth, and the possible presence of genotoxic effects in the species examined.

Since brodifacoum was not detected in the liver of the examined specimens despite its relatively “high” persistence and its potential for bioaccumulation [51], it can be hypothesized that there was no exposure of the marine organisms to brodifacoum residues despite there being confirmation of bait drift and bounce off cliffsides into the marine environment. There is insufficient evidence to claim that fish and other organisms, such as crustaceans and molluscs, could have traces of consumed pellets or accumulated brodifacoum residues. Perhaps, additional brodifacoum could run off into the marine system with rainfall bound to organic matter [52] or bait particulate and suspend in the water column where it could be directly or indirectly be consumed by benthic organisms [22]. The results indicate that the rat eradication method used in the Tavolara MPA, including the choice of the treatment season, the lapsed time, the quantity used, and the rather scarce quantity of bait dropped into the sea (unpublished data) water systems did not have adverse impacts on non-target marine species that are fundamental for the economy of the area. These results are consistent with observations from other island eradications using brodifacoum, in which the overall rate of residue detection was only 3.1% for the fish sampled [22]. In fact, after the aerial broadcast of pelleted bait, regular controls were conducted by scuba divers which did not register the presence of dead fish along the perimeter of the island. The lack of dead animals and brodifacoum residues in sampled fish suggest that the sublethal effects in exposed individual fish were very low. Further investigations are needed to gain a more thorough understanding of the effect of brodifacoum in the marine environment, including other non-target organisms.

## 5. Conclusions

The consequences of brodifacoum use for rat eradication in the terrestrial and marine environment should be considered. This study suggests that brodifacoum had no detectable impacts on the marine ecosystem following the use of brodifacoum to eradicate rats at Tavolara Island likely due to successful mitigation measures employed to minimize bait drift into the marine environment. The study confirmed that secondary brodifacoum exposure to people consuming fish harvested ten days after bait application was unlikely.

## Figures and Tables

**Table 1:** Mean ± S.D. (min–max) of the morphological parameters of the fish sampled at Tavolara Island.

Species	Weight (g)	Length (cm)	#Sample
Brown meagre (*Sciaena umbra*)	158.9 ± 15.7 (147.8–170)	24.75 ± 1.06 (24–25.5)	2
Painted comber (*Serranus scriba*)	86.32 ± 42.27 (33.7–153)	18.33 ± 3.37 (13–22)	6
Mediterranean rainbow wrasse (*Coris julis*)	30.68 ± 0	14 ± 0	1
Two-banded sea bream (*Diplodus vulgaris*)	123.03 ± 14.04 (105.9 ± 139)	18.9 ± 1.6 (17–21)	5
Comber (*Serranus cabrilla*)	22.5 ± 4.11 (19.59–25.4)	12 ± 1.41 (11–13)	2
Salema (*Sarpa salpa*)	435 ± 21.21 (420–450)	28 ± 0.71 (27.5–28.5)	2
Black seabream (*Spondyolisoma cantharus*)	400 ± 0	21 ± 0	1
Brown wrasse (*Labrus merula*)	800 ± 0	36.5 ± 0	1

**Table 2:** Mean frequency (‰) of each nuclear abnormality category (± S.D.) in peripheral erythrocytes of species sampled at Tavolara Island. Different letters indicate statistically significant differences (*p* < 0.05) using the ANOVA one-way with Tukey’s post-hoc test.

	Nuclear Abnormalities Categories				
Species	Lobed	Kidney-Shaped	Segmented	Micronuclei	#Sample
Brown meagre (*Sciaena umbra*)	26.5 ± 10.61 ^ab^	6 ± 0 ^c^	0 ± 0	1.5 ± 0.71 ^a^	2
Painted comber (*Serranus scriba*)	37 ± 26.34 ^ab^	2.83 ± 2.64 ^ab^	0.5 ± 0.84	0.83 ± 1.6 ^a^	6
Two-banded sea bream (*Diplodus vulgaris*)	15.5 ± 11.9 ^a^	0.25 ± 0.5 ^a^	0 ± 0	0 ± 0	4
Comber (*Serranus cabrilla*)	36 ± 0 ^b^	2 ± 0 ^b^	0 ± 0	0 ± 0	1
Salema (*Sarpa salpa*)	29.5 ± 30.41 ^ab^	2 ± 2.83 ^ab^	0 ± 0	1 ± 0 ^a^	2
Black seabream (*Spondyolisoma cantharus*)	46 ± 0 ^b^	1 ± 0 ^a^	0 ± 0	0 ± 0	1
Brown wrasse (*Labrus merula*)	91 ± 0 ^a^	7 ± 0 ^d^	0 ± 0	0 ± 0	1

## Data Availability

Data is contained within the article and Supplementary Materials.

## Supplementary Materials

The following supporting information can be downloaded at: https://www.mdpi.com/article/10.3390/life13020415/s1, Figure S1: HPLC chromatogram showing vitamin K and vitamin K 2, 3 epoxide standard solutions; Figure S2: HPLC chromatogram obtained by the standard brodifacoum solution (red) and by a sample (green).

## References

[1] Rattner B.A., Lazarus R.S., Elliott J.E., Shore R.F., van den Brink N. (2014). Adverse Outcome Pathway and Risks of Anticoagulant Rodenticides to Predatory Wildlife. Environ. Sci. Technol..

[2] Watt B.E., Proudfoot A.T., Bradberry S.M., Vale J.A. (2005). Anticoagulant Rodenticides. Toxicol Rev.

[3] Thijssen H.H.W., Janssen C.A.T., Mosterd J.J. (1989). Warfarin Resistance: Biochemical Evaluation of a Warfarin-Resistant Wild Brown Rat. Biochem. Pharmacol..

[4] Kotthoff M., Rüdel H., Jürling H., Severin K., Hennecke S., Friesen A., Koschorreck J. (2019). First Evidence of Anticoagulant Rodenticides in Fish and Suspended Particulate Matter: Spatial and Temporal Distribution in German Freshwater Aquatic Systems. Environ. Sci. Pollut. Res..

[5] Eason C.T., Murphy E.C., Wright G.R.G., Spurr E.B. (2002). Assessment of Risks of Brodifacoum to Non-Target Birds and Mammals in New Zealand. Ecotoxicology.

[6] Fasco M.J., Principe L.M. (1982). R-and S-Warfarin inhibition of vitamin K and vitamin K 2, 3-epoxide reductase activities in the rat. J. Biol. Chem..

[7] Wallin R., Martin L.F. (1985). Vitamin K-Dependent Carboxylation and Vitamin K Metabolism in Liver. Effects of Warfarin. J. Clin. Investig..

[8] Dubock A.C., Kaukeinen D.E., Carolina N. Brodifacoum (Talon™ rodenticide), a novel concept. Proceedings of the Vertebrate Pest Conference.

[9] Hooker S., Innes J. (1995). Ranging Behaviour of Forest-dwelling Ship Rats, *Rattus Rattus,* and Effects of Poisoning with Brodifacoum. N. Z. J. Zool..

[10] Erickson W.A., Urban D.J. (2004). Potential Risks of Nine Rodenticides to Birds and Nontarget Mammals: A Comparative Approach.

[11] Lambert O., Pouliquen H., Larhantec M., Thorin C., L’Hostis M. (2007). Exposure of Raptors and Waterbirds to Anticoagulant Rodenticides (Difenacoum, Bromadiolone, Coumatetralyl, Coumafen, Brodifacoum): Epidemiological Survey in Loire Atlantique (France). Bull. Environ. Contam. Toxicol..

[12] Fournier-Chambrillon C., Berny P.J., Coiffier O., Barbedienne P., Dassé B., Delas G., Galineau H., Mazet A., Pouzenc P., Rosoux R. (2004). Evidence of secondary poisoning of free-ranging riparian mustelids by anticoagulant rodenticides in France: Implications for conservation of european mink (Mustela lutreola). J. Wildl. Dis..

[13] Stone W.B., Okoniewski J.C., Stedelin J.R. (1999). Poisoning of wildlife with anticoagulant rodenticides in New York. J. Wildl. Dis..

[14] Stone W.B., Okoniewski J.C., Stedelin J.R. (2003). Anticoagulant Rodenticides and Raptors: Recent Findings from New York, 1998-2001. Bull. Environ. Contam. Toxicol..

[15] Shore R.F., Birks J.D.S., Afsar A., Wienburg C.L., Kitchener A.C. (2003). Spatial and Temporal Analysis of Second-Generation Anticoagulant Rodenticide Residues in Polecats (Mustela Putorius) from throughout Their Range in Britain, 1992–1999. Environ. Pollut..

[16] Alomar H., Chabert A., Coeurdassier M., Vey D., Berny P. (2018). Accumulation of Anticoagulant Rodenticides (Chlorophacinone, Bromadiolone and Brodifacoum) in a Non-Target Invertebrate, the Slug, Deroceras Reticulatum. Sci. Total. Environ..

[17] Sánchez-Barbudo I.S., Camarero P.R., Mateo R. (2012). Primary and Secondary Poisoning by Anticoagulant Rodenticides of Non-Target Animals in Spain. Sci. Total. Environ..

[18] Wiens J.D., Dilione K.E., Eagles-Smith C.A., Herring G., Lesmeister D.B., Gabriel M.W., Wengert G.M., Simon D.C. (2019). Anticoagulant Rodenticides in Strix Owls Indicate Widespread Exposure in West Coast Forests. Biol. Conserv..

[19] Fisher P., Griffiths R., Speedy C., Broome K. (2010). Environmental Monitoring for Brodifacoum Residues after Aerial Application of Baits for Rodent Eradication. Proc. Vertebr. Pest Conf..

[20] Howald G., Donlan C.J., Faulkner K.R., Ortega S., Gellerman H., Croll D.A., Tershy B.R. (2010). Eradication of Black Rats Rattus Rattus from Anacapa Island. Oryx.

[21] Dowding J.E., Lovegrove T.I.G., Ritchie J., Kast S.N., Puckett M. (2006). Mortality of northern New Zealand dotterels (Charadrius obscurus aquilonius) following an aerial poisoning operation. Notornis.

[22] Masuda B.M., Fisher P., Beaven B. (2015). Residue Profiles of Brodifacoum in Coastal Marine Species Following an Island Rodent Eradication. Ecotoxicol. Environ. Saf..

[23] Primus T., Wright G., Fisher P. (2005). Accidental Discharge of Brodifacoum Baits in a Tidal Marine Environment: A Case Study. Bull. Environ. Contam. Toxicol..

[24] Pitt W.C., Berentsen A.R., Shiels A.B., Volker S.F., Eisemann J.D., Wegmann A.S., Howald G.R. (2015). Non-Target Species Mortality and the Measurement of Brodifacoum Rodenticide Residues after a Rat (Rattus Rattus) Eradication on Palmyra Atoll, Tropical Pacific. Biol. Conserv..

[25] Siers S., Shiels A., Volker S., Rex K. (2020). Brodifacoum Residues in Fish Three Years after an Island-Wide Rat Eradication Attempt in the Tropical Pacific. MBI.

[26] Broome K., Golding C., Brown K., Corson P., Bell P. (2017). Rat Eradication Using Aerial Baiting Current Agreed Best Practice Used in New Zealand.

[27] Christensen T.K., Lassen P., Elmeros M. (2012). High Exposure Rates of Anticoagulant Rodenticides in Predatory Bird Species in Intensively Managed Landscapes in Denmark. Arch. Environ. Contam. Toxicol..

[28] Sherman P.A., Sander E.G. (1981). Vitamin K Epoxide Reductase: Evidence That Vitamin K Dihydroquinone Is a Product of Vitamin K Epoxide Reduction. Biochem. Biophys. Res. Commun..

[29] Jakob E., Elmadfa I. (1996). Application of a Simplified HPLC Assay for the Determination of Phylloquinone (Vitamin K1) in Animal and Plant Food Items. Food Chem..

[30] Pacheco M., Santos M.A. (1997). Induction of EROD Activity and Genotoxic Effects by Polycyclic Aromatic Hydrocarbons and Resin Acids on the Juvenile Eel (*Anguilla anguilla* L.). Ecotoxicol. Environ. Saf..

[31] Mosterd J.J., Thijssen H.H.W. (1991). The Long-Term Effects of the Rodenticide, Brodifacoum, on Blood Coagulation and Vitamin K Metabolism in Rats. Br. J. Pharmacol..

[32] Ostermeyer U., Schmidt T. (2001). Determination of Vitamin K in the Edible Part of Fish by High-Performance Liquid Chromatography. Eur. Food Res. Technol..

[33] Rattner B.A., Horak K.E., Warner S.E., Johnston J.J. (2010). Acute Toxicity of Diphacinone in Northern Bobwhite: Effects on Survival and Blood Clotting. Ecotoxicol. Environ. Saf..

[34] Sage M., Fourel I., Cœurdassier M., Barrat J., Berny P., Giraudoux P. (2010). Determination of Bromadiolone Residues in Fox Faeces by LC/ESI-MS in Relationship with Toxicological Data and Clinical Signs after Repeated Exposure. Environ. Res..

[35] Kawatsu H. (1986). Clotting Time of Common Carp Blood. NIPPON SUISAN GAKKAISHI.

[36] Yildiz H.Y. (2009). Reference Biochemical Values for Three Cultured Sparid Fish: Striped Sea Bream, Lithognathus Mormyrus; Common Dentex, Dentex Dentex; and Gilthead Sea Bream, *Sparus aurata*. Comp. Clin. Pathol..

[37] Bolognesi C., Cirillo S. (2014). Genotoxicity Biomarkers in Aquatic Bioindicators. Curr. Zool..

[38] Caliani I., Poggioni L., D’Agostino A., Fossi M.C., Casini S. (2019). An Immune Response-Based Approach to Evaluate Physiological Stress in Rehabilitating Loggerhead Sea Turtle. Veter-Immunol. Immunopathol..

[39] Costa P.M., Lobo J., Caeiro S., Martins M., Ferreira A.M., Caetano M., Vale C., DelValls T.Á., Costa M.H. (2008). Genotoxic Damage in Solea Senegalensis Exposed to Sediments from the Sado Estuary (Portugal): Effects of Metallic and Organic Contaminants. Mutat. Res./Genet. Toxicol. Environ. Mutagen..

[40] Maceda-Veiga A., Monroy M., Viscor G., De Sostoa A. (2010). Changes in Non-Specific Biomarkers in the Mediterranean Barbel (Barbus Meridionalis) Exposed to Sewage Effluents in a Mediterranean Stream (Catalonia, NE Spain). Aquat. Toxicol..

[41] Marques A., Custódio M., Guilherme S., Gaivão I., Santos M.A., Pacheco M. (2014). Assessment of Chromosomal Damage Induced by a Deltamethrin-Based Insecticide in Fish (*Anguilla anguilla* L.)—A Follow-up Study upon Exposure and Post-Exposure Periods. Pestic. Biochem. Physiol..

[42] Marques A., Rego A., Guilherme S., Gaivão I., Santos M.A., Pacheco M. (2016). Evidences of DNA and Chromosomal Damage Induced by the Mancozeb-Based Fungicide Mancozan® in Fish (*Anguilla anguilla* L.). Pestic. Biochem. Physiol..

[43] Pacheco M., Santos M.A. (1999). Biochemical and Genotoxic Responses of Adult Eel (*Anguilla anguilla* L.) to Resin Acids and Pulp Mill Effluent: Laboratory and Field Experiments. Ecotoxicol. Environ. Saf..

[44] Pacheco M., Santos M.A. (2002). Biotransformation, Genotoxic, and Histopathological Effects of Environmental Contaminants in European Eel (*Anguilla anguilla* L.). Ecotoxicol. Environ. Saf..

[45] Teles M., Pacheco M., Santos M.A. (2005). *Sparus aurata* L. Liver EROD and GST Activities, Plasma Cortisol, Lactate, Glucose and Erythrocytic Nuclear Anomalies Following Short-Term Exposure Either to 17β-Estradiol (E2) or E2 Combined with 4-Nonylphenol. Sci. Total. Environ..

[46] Ware K.M., Feinstein D.L., Rubinstein I., Weinberg G., Rovin B.H., Hebert L., Muni N., Cianciolo R.E., Satoskar A.A., Nadasdy T. (2015). Brodifacoum Induces Early Hemoglobinuria and Late Hematuria in Rats: Novel Rapid Biomarkers of Poisoning. Am. J. Nephrol..

[47] Oliveira M., Pacheco M., Santos M.A. (2007). Cytochrome P4501A, Genotoxic and Stress Responses in Golden Grey Mullet (Liza Aurata) Following Short-Term Exposure to Phenanthrene. Chemosphere.

[48] Maria V.L., Correia A.C., Santos M.A. (2003). Genotoxic and Hepatic Biotransformation Responses Induced by the Overflow of Pulp Mill and Secondary-Treated Effluents on *Anguilla anguilla* L.. Ecotoxicol. Environ. Saf..

[49] Rodrigues S., Antunes S.C., Correia A.T., Golovko O., Žlábek V., Nunes B. (2019). Assessment of Toxic Effects of the Antibiotic Erythromycin on the Marine Fish Gilthead Seabream (*Sparus aurata* L.) by a Multi-Biomarker Approach. Chemosphere.

[50] Gusso-Choueri P.K., Choueri R.B., Santos G.S., de Araújo G.S., Cruz A.C.F., Stremel T., de Campos S.X., Cestari M.M., Ribeiro C.A.O., de Sousa Abessa D.M. (2016). Assessing Genotoxic Effects in Fish from a Marine Protected Area Influenced by Former Mining Activities and Other Stressors. Mar. Pollut. Bull..

[51] Regnery J., Parrhysius P., Schulz R.S., Möhlenkamp C., Buchmeier G., Reifferscheid G., Brinke M. (2019). Wastewater-Borne Exposure of Limnic Fish to Anticoagulant Rodenticides. Water Res..

[52] Housenger J., Melendez J.L. (2012). Risks of Brodifacoum Use to the Federally Threatened Alameda Whipsnake (Masticophis lateralis euryxanthus), and the Federally Endangered Salt Marsh Harvest Mouse (Reithrodontomys raviventris) and San Joaquin Kit Fox (Vulpes macrotis mutica).

