# Supplementary material for: Brodifacoum Levels and Biomarkers in Coastal Fish Species following a Rodent Eradication in an Italian Marine Protected Area: Preliminary Results
Source: Life (Basel). 2023 Feb 2;13(2):415. doi: 10.3390/life13020415 (PMC9966337; doi:10.3390/life13020415)
Supplement: Supplementary file 1 [file life-13-00415-s001.zip › life-2141389-supplementary.pdf]

Brodifacoum levels and biomarkers in coastal fish species following a rodent eradication in an Italian Marine Protected Area: preliminary results

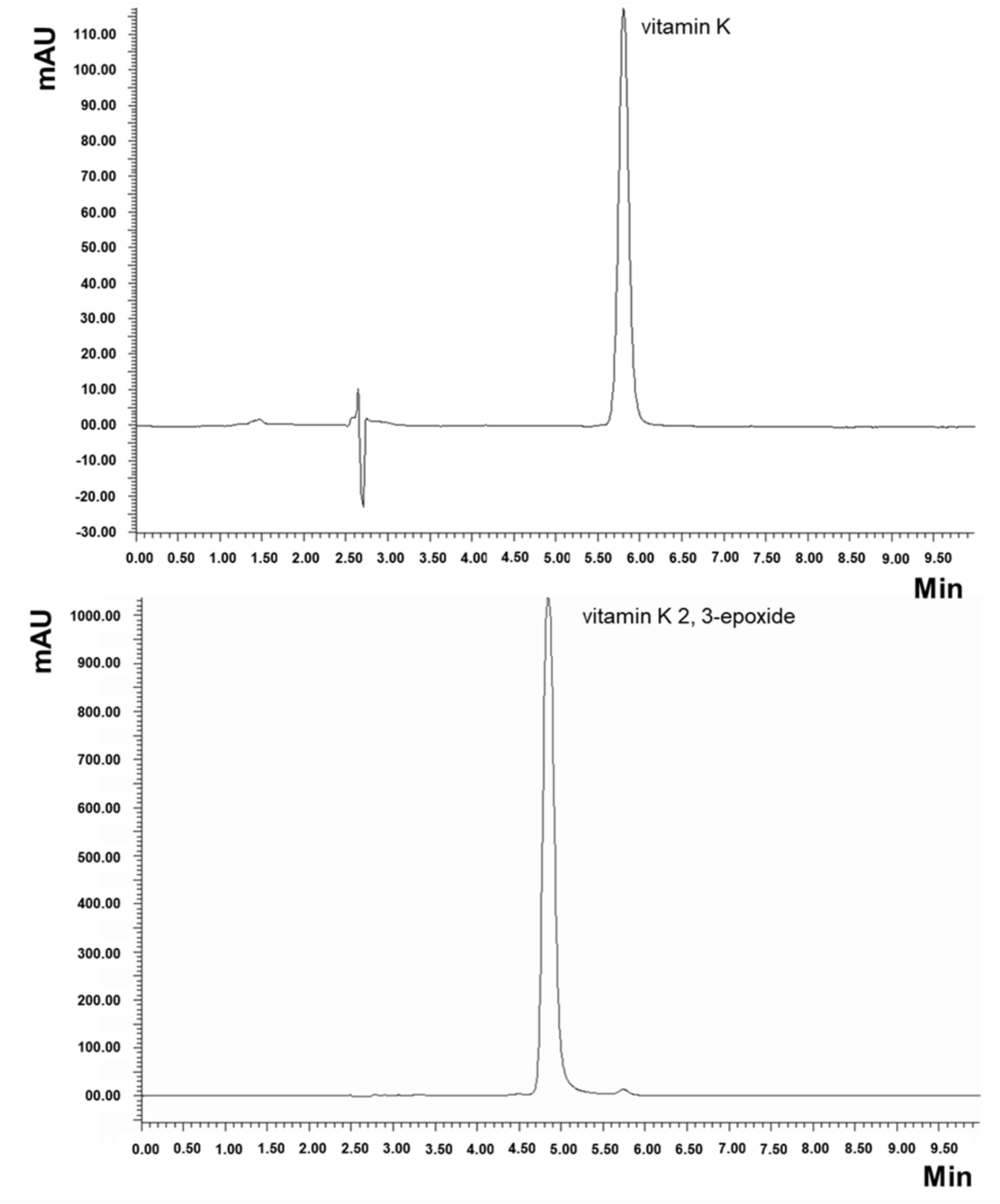

Figure S1: HPLC chromatogram showing vitamin K and vitamin K 2, 3 epoxide standard solutions.

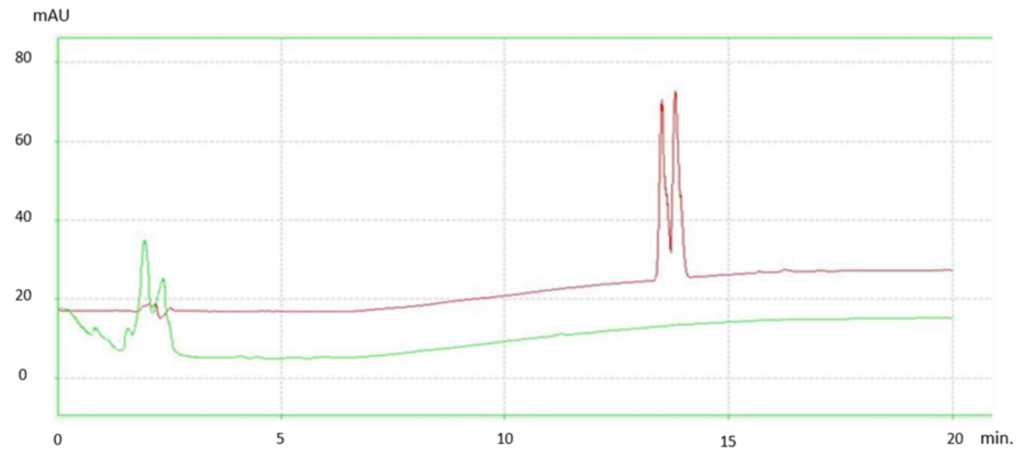

Figure S2: HPLC chromatogram obtained by the standard brodifacoum solution (red) and by a sample (green).
